# Phenolic Compounds, Antioxidant and Antimicrobial Activities of *Punica granatum* L. Fruit Extracts

**DOI:** 10.3390/molecules31020334

**Published:** 2026-01-19

**Authors:** Mijat Božović, Vanja Tadić, Alessandra Oliva, Milan Mladenović, Roberta Astolfi, Rino Ragno

**Affiliations:** 1 Faculty of Science and Mathematics, University of Montenegro, Džordža Vašingtona bb, 81000 Podgorica, Montenegro; mijatboz@ucg.ac.me; 2 Institute of Medicinal Plants Research Dr. Josif Pančić, Tadeuša Koščuška 1, 11000 Belgrade, Serbia; vtadic@mocbilja.rs; 3 Department of Public Health and Infectious Diseases, Sapienza University or Rome, Piazzale Aldo Moro 5, 00185 Rome, Italy; alessandra.oliva@uniroma1.it; 4 Kragujevac Center for Computational Biochemistry, Faculty of Science, University of Kragujevac, Radoja Domanovića 12, 34000 Kragujevac, Serbia; milan.mladenovic@pmf.kg.ac.rs; 5 Rome Center for Molecular Design, Department of Drug Chemistry and Technology, Sapienza University of Rome, Piazzale Aldo Moro 5, 00185 Rome, Italy; roberta.astolfi@uniroma1.it

**Keywords:** pomegranate, juice extract, pericarp extract, punicalagin isomers, bioactivity, antioxidant, MRSA, MSSA, CRAB

## Abstract

Pomegranate is valued for its abundant polyphenolic content and its capacity to promote health. In this study, pomegranate juice or pericarp extracts from two Mediterranean regions (Montenegro and Italy) were systematically and comparatively evaluated for the first time with respect to their polyphenolic composition, antioxidant capacity, and antimicrobial activity. The extraction of juice extracts was accomplished by means of the Kutscher–Steudel liquid–liquid extraction technique, which was employed to selectively recover phenolics. In contrast, the extraction of pericarp extracts from the solid matrix was achieved via Soxhlet extraction. A thorough high-performance liquid chromatography (HPLC) analysis was conducted to identify and quantify the major phenolic compounds present in the sample. This analysis revealed the presence of ellagitannin punicalagin isomers, with concentrations reaching up to 254.75 mg/g of the sample, as well as ellagic acid and gallic acid. The antioxidant potential of the samples was assessed using the antioxidant activity index (AAI) from the 2,2-diphenyl-1-picrylhydrazyl (DPPH) test and by a ferric reducing antioxidant power (FRAP) assay. Juice extracts demonstrated a range of activity levels, with AAI values ranging from 0.17 to 2.12 and FRAP values ranging from 2.49 to 19.41 mmol Fe^2+^/g. In contrast, pericarp extracts exhibited notably higher activity, with AAI values ranging from 0.18 to 27.57 and FRAP values ranging from 2.99 to 372.17 mmol Fe^2+^/g. This study demonstrates the markedly higher functional potential of pericarp extracts compared to juice extracts by linking detailed phenolic profiles with bioactivity data. Antimicrobial testing, inclusive of the determination of minimum bactericidal concentration (MBC), demonstrated that certain pericarp extracts manifested bactericidal properties at low concentrations against selected clinically pertinent strains, including methicillin-resistant *Staphylococcus aureus* (0.109% *p*/*v*), methicillin-sensitive *S. aureus* (0.109% *p*/*v*), carbapenem-resistant *Acinetobacter baumannii* (0.109% *p*/*v*), and *Escherichia coli* (0.563% *p*/*v*). *Candida albicans* and *Klebsiella pneumoniae* strains exhibited minimal sensitivity to these extracts. The findings indicate that pomegranate pericarp is a valuable by-product, and they demonstrate the potential of both juice and pericarp extracts as functional ingredients.

## 1. Introduction

The field of nutraceuticals has seen a surge in interest, driven by the potential nutritional, safety, and therapeutic benefits they offer. In addition, a wide array of biological activities, including antioxidant, antimicrobial, antihypertensive, anticancer, and antigenotoxic properties, have been repeatedly observed to be associated with bioactive compounds derived from food by-products [1]. These compounds, frequently designated as functional ingredients, constitute a diverse group of secondary metabolites, with phenol- containing mixtures being the most prominent [2]. Nonetheless, the recovery of the subjects from food and/or by-products is significantly impacted by the extraction technique [3].

*Punica granatum* L. (pomegranate) is among the earliest cultivated plants, distinguished by its edible fruits, which have gained significant popularity in the food industry. Despite the absence of consensus regarding its precise origins, the species’ native range is believed to extend from north-eastern Turkey to western and northern Pakistan [4]. The subject of this study is a deciduous and spiny shrub or small tree from the *Lythraceae* family. It grows to between 5 and 10 m in height, and its leaves are oblong–lanceolate and glabrous. The leaves are oppositely arranged. The flowers, which range in color from orange-red to crimson, are 5–8-merous and possess hypanthia, as well as many-celled inferior ovaries and numerous stamens. The fruits are classified as globose berries, with an average diameter ranging from 5 to 8 cm. They possess a leathery, predominantly reddish-brown exocarp (rind) and an inner, spongy mesocarp (albedo) that terminates in thin, whitish–yellow endocarp partitions. These partitions form asymmetric chambers that contain the seeds. The seeds are characterized by their numerous, angular, and obpyramidal form. They exhibit a variety of colors, including purple, pink, and white, and are distinguished by their juicy, sarcotestal layers, which are often erroneously referred to as arils [5].

The pomegranate is a fruit that has been cultivated in temperate and subtropical regions for millennia. In addition to its historical applications, the consumption of pomegranate fruits has been linked to a variety of health benefits, which have been the focus of extensive research in recent years [6]. These health-promoting effects of pomegranate have also been documented in traditional phytotherapy. A review of the literature suggests that these effects are primarily attributable to its polyphenol content and antioxidant activity [7]. While the seeds are the most commonly consumed portion of the pomegranate, various inedible parts have been reported to contain a high concentration of bioactive compounds. These compounds have been utilized in the treatment of various pathological conditions within traditional medicinal practices. For instance, pomegranate bark is employed in the treatment of intestinal parasites, dysentery, and diarrhea [8], while the leaves are utilized to address various infections, fever, influenza, and pneumonia [9]. The juice of the pomegranate, obtained by squeezing the seeds and/or whole fruits of the plant, has attracted particular research interest due to its rich bioactive components content and multitude of health benefits that extend beyond basic nutrition [10]. However, industrial application has led to an increase in the production of waste material, such as pericarp (fruit peel) and seed remains, which has gradually prompted exploration of these pomegranate parts.

As demonstrated in previous studies, the pomegranate pericarp is characterized by a high concentration of phenolic compounds, with hydrolysable tannins representing the predominant category. These compounds have been shown to possess significant antioxidant, antimicrobial, antiproliferative, and anti-inflammatory properties [11]. The seeds are also abundant in phytochemicals, including unsaturated fatty acids (e.g., punicic acid), which exhibit a variety of pharmacological effects, such as antitumor and anti-osteoporosis properties, as well as efficacy against diabetes and obesity [12]. Furthermore, the increased industrial interest in pomegranates has led to an increase in the consumption of other pomegranate products, including alcoholic beverages, syrups, jams, and jellies [13]. In light of the dearth of standardized methodologies for the extraction of bioactive materials, and given the acknowledgment that the chemical composition and bioavailability of metabolites can be influenced by food processing [14], the objective of this study was to comparatively evaluate the polyphenolic composition and related bioactivities of pomegranate juice and pericarp extracts obtained from cultivated and wild fruits collected in Montenegro and Italy. The objective of the present study was to identify which extracts exhibited the highest antioxidant and antimicrobial potential. The findings establish a basis for additional research on the use of pomegranate in pharmaceuticals and cosmeceuticals.

## 2. Results

### 2.1. Extraction Yields

The liquid–liquid extraction procedure applied to three juice samples yielded between 0.64 and 6.26 g of extract per 100 mL of juice (see Table 1). Among the solvents utilized in this study, ethyl acetate yielded a significantly higher amount than diethyl ether, with variances exceeding fivefold in certain instances (e.g., JE1 and JE4). The incorporation of 1% (*v*/*v*) isopropanol into ethyl acetate resulted in a modest alteration of its polarity. This modification enhanced the solubility of specific compounds and augmented its capacity to extract a broader array of polar and semi-polar bioactive compounds. Consequently, this combination yielded even greater results. A comparison of cultivated and wild pomegranates revealed that the latter produced higher extract yields, ranging from 2.58 to 6.26 g per 100 mL. It is noteworthy that when ethyl acetate and its mixture with isopropanol were utilized, the Montenegrin cultivar yielded a lower quantity of extract compared to the Italian cultivar. Nevertheless, an antithetical pattern was observed in the case of diethyl ether utilized as the solvent.

The solid–liquid extraction procedure applied to the pericarp samples yielded between 0.16% and 63.51% (*w*/*w*), as shown in Table 2. A subsequent comparison of the separate and successive extractions revealed no significant differences. Among the various solvents employed, methanol demonstrated the highest extracting power, followed by ethyl acetate. In contrast, hexane and dichloromethane exhibited suboptimal efficiency. In this case, it is noteworthy that higher yields were achieved with the cultivated pomegranate from Montenegro (e.g., PE8, PE11, and PE23). However, this cannot be definite as a general rule, as a higher yield was obtained from the pericarp of the Italian cultivar when dichloromethane was used (PE4 and PE16), thus confirming the importance of several parameters influencing both yields and compositions.

### 2.2. Polyphenolic Constituents

A total of nine juice extracts and twenty pericarp extracts were subjected to analysis to determine their polyphenolic content. A variety of phenolic acids, including gallic and *p*-hydroxycinnamic acids, as well as ellagic acid and the ellagitannins punicalin and *α*- and *β*-punicalagins, were detected at varying concentrations. The study also encompassed the detection of anthocyanin compounds. Cyanidin chloride and malvidin-3-*O*-glucoside were identified through fingerprinting and were solely reported for three juice extracts (JE2, JE3, and JE6) (Table 3). Due to the inadequate quantities of pericarp extracts, specifically those extracted using hexane and dichloromethane (PE1, PE4, PE14, and PE17), they were excluded from further consideration.

The analysis revealed that several of the juice extracts (JE4, JE5, JE6, JE7) were particularly rich in punicalagins, with the Italian cultivar extracts exhibiting notably higher concentrations (up to 11.7 mg/g). In the present study, the extraction efficiency of ethyl acetate, both in isolation and in conjunction with isopropanol, was found to be notably superior to that of diethyl ether. The highest concentrations of ellagic acid were detected in the wild sample extracts (JE3, JE6, JE9), while JE8 (Montenegrin cultivar juice extracted with isopropanol and ethyl acetate) furnished a significantly higher amount of gallic acid (3.81 mg/g).

For the pericarp extracts, a notably high punicalagins content was observed in the methanolic extracts (207.58 to 254.75 mg/g). As indicated by the findings of this study, ethyl acetate demonstrated notable extraction efficacy, with a maximum of 18.81 mg/g (mg/g) observed in the PE8 extract. In contrast, dichloromethane, and to a greater extent hexane, exhibited suboptimal extraction capabilities for these ellagitannins. The same pattern was observed for punicalin, with concentrations ranging from 1.96 to 5.72 mg/g (mg/g) in the methanolic extracts. However, the PE20 extract, obtained with ethyl acetate, exhibited a higher concentration of this ellagitannin, reaching up to 9.22 mg/g.

In the context of gallic and ellagic acids, a comparative analysis was conducted to assess the extracting capacity of various extraction agents. The results indicated that ethyl acetate exhibited a marginally superior extracting capacity compared to methanol. A notable finding was the abundance of phenolic compounds in the pericarp samples from Montenegro, both cultivated and wild, with gallic acid ranging from 3.42 to 9.21 mg/g and ellagic acid from 20.83 to 30.03 mg/g. While *p*-hydroxycinnamic acid was detected in a limited number of extracts, the efficacy of hexane and dichloromethane in extracting this phenolic acid is particularly noteworthy. For instance, the hexane extracts PE3 and PE15 (from the wild sample) contained 2.23 and 4.47 mg/g, respectively, while the dichloromethane extract PE16 (from the Italian cultivar) contained as much as 9.93 mg/g.

### 2.3. Antioxidant Activity

A variety of assays can be utilized to assess antioxidant potential, each exhibiting a distinct mechanism of action. Consequently, it is strongly recommended to perform different antioxidant measurements to comprehensively assess the extracts activity. In the present study, the activity of either pomegranate juice or pericarp extracts was evaluated using two in vitro tests: the 2,2-diphenyl-1-picrylhydrazyl (DPPH) assay, which assesses proton-radical scavenging activity, and the ferric reducing antioxidant power (FRAP) method, which evaluates the ability of antioxidants to reduce ferric (Fe*^3+^*) ions to ferrous (Fe*^2+^*) ions. However, a lack of statistically significant differences in antioxidant capacity was observed between the applied methods.

A total of nine juice extracts and fifteen pericarp extracts were subjected to analysis to determine their antioxidant capacity. The activity estimated by DPPH was expressed in terms of IC_50_ (µg/mL) (Table 4). According to the calculated AAI values, the juice extracts obtained using diethyl ether exhibited weak antioxidant capacity (J1–J3), while those obtained with ethyl acetate and its combination with isopropanol exhibited moderate (J4, J5, J7, J9), high (J6), or even very strong (J8) activity. Conversely, all pericarp extracts obtained with ethyl acetate and methanol exhibited remarkably robust antioxidant activity, with AAI values ranging from 3.14 to 27.57. Due to the suboptimal yield, only a subset of the extracts obtained with hexane and dichloromethane were subjected to testing (PE1, PE5, and PE18). However, a weak antioxidant activity was observed, with AAI values ranging from 0.18 to 0.19. The ferric reducing ability (FRAP) values were expressed in mmol Fe^2+^ per g extract. The reducing power capacities exhibited a high degree of concordance with the DPPH assay results, with values ranging from 2.49 to 19.41 mmol Fe^2+^/g and from 2.99 to 372.17 mmol Fe^2+^/g for the extracts obtained from juice and pericarp, respectively (see Table 4 for details).

### 2.4. Antimicrobial Activity

A total of five juice extracts and eight pericarp extracts were subjected to analysis to assess their antimicrobial efficacy. As demonstrated in Table 5, the activity exhibited variability depending on the tested pathogens. Specifically, none of the pomegranate extracts demonstrated activity against *Candida albicans* (CA), and the activity was negligible against all Enterobacterales, including either carbapenem-susceptible or carbapenem re-sistant *Klebsiella pneumoniae* clinical strain (KPC). Conversely, the most pronounced activity was observed against methicillin-resistant *Staphylococcus aureus* (MRSA) and methicillin-susceptible *Staphylococcus aureus* (MSSA) strains, as well as the carbapenem-resistant *Acinetobacter baumannii* (CRAB) strain.

The most potent extracts against MSSA and MRSA exhibited similar activity profiles, including JE6 (0.159/0.159% *p*/*v* for MSSA/MRSA, respectively), PE8 (1.248/1.248% *p*/*v*), PE10 (1.271/1.271% *p*/*v*), PE23 (0.337/0.168% *p*/*v*), and PE24 (0.109/0.109% *p*/*v*). In contrast, PE21 demonstrated higher potency against MSSA (0.952% *p*/*v*) than MRSA (>1.904% *p*/*v*). The samples PE23 and PE24 exhibited the strongest inhibitory activity (0.674% and 0.109% *p*/*v*, respectively) against CRAB, comparable to their activity against MSSA and MRSA strains. Furthermore, it was demonstrated that JE2, JE3, JE6, and JE8 exhibited bactericidal properties at concentrations of 0.308%, 0.252%, 0.638%, and 0.805% *p*/*v*, respectively. It was observed that certain samples demonstrated activity against *E. coli*, including the juice extracts (JE2, JE6, and JE8), as well as the pericarp extract PE24. The minimum bactericidal concentration (MBC) values of these samples ranged from 0.563 to 0.877% *p*/*v*.

## 3. Discussion

### 3.1. Extraction Yields and Identified Phenolic Constituents

With regard to the collective performance of solvents in extraction processes, the observed variations can be ascribed to differences in polarity and solubility [15,16]. It has been established that the polarity of solvents plays a pivotal role in the extraction process. Variations in polarity influence the solvent’s ability to dissolve specific compounds, thereby affecting the expression of their bioactivities [17]. For extractions performed on juices, the yields followed a predictable trend, with ethyl acetate yielding higher than isopropanol and diethyl ether. Diethyl ether, being the least polar solvent, extracted the smallest number of bioactive compounds. This is likely due to the fact that it favors nonpolar or weakly polar constituents. Moderately polar ethyl acetate proved to be a more efficacious extraction agent, facilitating the recovery of a more extensive array of bioactive compounds. The incorporation of isopropanol led to a notable enhancement in the polarity of ethyl acetate, thereby increasing the solubility of more polar compounds and culminating in the attainment of the highest yield. For the pericarp samples, the yields followed the following sequence: methanol > ethyl acetate > dichloromethane > hexane. Methanol, being the most polar solvent, revealed the highest extraction efficacy, likely due to its ability to dissolve a wide range of polar compounds. In addition, ethyl acetate, which possesses intermediate polarity, also demonstrated a good extraction power. However, its extraction efficiency was significantly lower than that of methanol. Conversely, the weakly polar dichloromethane and nonpolar hexane exhibited lower extraction ability but with a selectivity in predominantly extract lipophilic compounds, which are present in limited amounts in the pomegranate pericarp.

The extraction yields are largely consistent with the literature on the subject. As indicated in the literature, comparable solvent efficiencies were reported for the pericarp extracts of Indian origin [18]. The highest yield was achieved using methanol (37.8%), followed by ethyl acetate (16.5%) and dichloromethane (5.9%), whereas the lowest was with hexane (2.1%). However, it has been observed that, while the yields of the methanolic extracts in the present study were higher, especially in the cultivar samples, the extraction yields of ethyl acetate, dichloromethane, and hexane extracts were considerably lower. In contrast, another study on the pericarp material of Indian origin reported that methanol (23.56%) and ethyl acetate (2.2%) were less efficient [19], and similar yields were obtained with the Egyptian material (35.4% and 1% for methanolic and ethyl acetate extracts, respectively) [20]. The study on the Iranian pomegranate sample demonstrated that the extraction power was significantly influenced by the methanol concentration. The use of 50% methanol resulted in only 18.9% of the extract [21]. Conversely, it is widely acknowledged that the selection of an appropriate extraction technique and the optimization of extraction parameters are pivotal factors in the recovery of valuable bioactive metabolites [22]. For this reason, extraction methods are frequently compared to ascertain the most optimal solution. A study [23] conduced with Soxhlet and ultrasound-assisted extractions demonstrated the superiority of the latter method, further emphasizing the efficacy of methanol (up to 51% and 39.4% for ultrasound and Soxhlet extraction, respectively).

Polyphenols are the predominant phytochemicals in pomegranate fruits, primarily represented by hydrolysable tannins (including gallotannins and ellagitannins, with unique gallagyl esters such as punicalagins). These compounds are mainly concentrated in the pericarp [24]. Punicalagin *α* and *β* isomers are frequently regarded as the primary bioactive constituents of pomegranate [25]. Despite their limited abundance in the seed sarcotesta (pulp), these particular ellagitannins have the potential to be present in the juice, contingent upon the utilized processing technology. This phenomenon can be attributed, in large part, to the prevalence of whole fruits being utilized for extraction rather than solely the pulpy seeds [26], as evidenced by the present study. In addition to ellagitannins and ellagic acid derivatives, pomegranate juice is a notable source of anthocyanins, which are predominantly composed of delphinidin and cyanidin derivatives. These anthocyanins are widely regarded as essential quality compounds [27]. However, flavanols, including procyanidins, were reported as the most abundant constituents [28]. The fruit pericarp, which is typically regarded as a by-product of the food industry, is being recognized as a significant source of bioactive phenolics [29]. The fruit pericarp comprises approximately 40% of the total fruit, and numerous studies have documented substantial quantities of hydrolysable tannins, phenolic acids, anthocyanins, and other flavonoids [22].

A substantial body of research has been conducted on the consumption of pomegranate juice, which is the most widely consumed form of this fruit. Furthermore, it is important to note that juice is concentrated, and lyophilized residues are often considered as extracts [30]. However, to the best of the authors’ knowledge, no data are available on extracts obtained directly from juice using a liquid–liquid extraction procedure. With regard to pericarp extracts, a variety of methodologies and methodological approaches have been employed, frequently yielding inadequate or even incongruent data. Consequently, a comparison of the results with previously published data is challenging, and in some cases, it is not possible. However, a study [17] provided valuable insights into sarcotestal ingredients. The study involved extracting the sarcotestal layers from the seeds and manually separating them. The results revealed varying amounts of ellagic acid (up to 34.5 mg/g), gallic acid (up to 3.37 mg/g), and punicalagins (up to 2.07 mg/g), depending on the solvent used. Nevertheless, the utilization of disparate solvents from those employed in the present study was observed, with ethanol emerging as the most efficacious.

The analysis of the juice extracts revealed the presence of punicalagins *α* and *β*, with the cultivar sample from Italy exhibiting particularly high concentrations. Ellagic acid was also detected in significant amounts, particularly in both cultivated and wild Montenegrin samples. Furthermore, an antithetical pattern of the distribution of these compounds was observed in the aforementioned samples (JE5, JE6, JE8, JE9). It is imperative to consider the instability of punicalagins, classified as hydrolyzable tannins, particularly when subjected to suboptimal storage conditions. This instability can lead to a series of reactions, including hydrolysis, which in turn may result in the formation of ellagic acid and other metabolites, as previously documented [31]. Conversely, the absence of anthocyanins in the extracts, despite their prevalence in the juice, can be ascribed to their instability and/or thermal sensitivity during the extraction process. In addition to the formation of destabilized aglycones as degradation products and their further spontaneous transformation into chalcones by opening their heterocyclic rings, anthocyanins also tend to form complex derivatives with tannins. This transformation is often accompanied by a color change, and it is particularly prevalent in juices derived from whole pomegranate fruits, which possess a high tannin content derived from the pericarp [32]. As these complex compounds are not initially present in pomegranate juice but are eventually formed, their extraction from the juice enhances the total polyphenol content and potentially influences bioactivity.

A comparison of the results for the pericarp extracts with existing literature data revealed some similarities. For instance, the analyses of the methanolic extracts of three Moroccan pomegranate samples demonstrated higher punicalagin content, with concentrations ranging from 200.21 to 216.36 and from 128.57 to 154.94 mg/g for the *α* and *β* isomers, respectively. Additionally, ellagic acid (32.14–35.00 mg/g) and gallic acid (1.86–2.14 mg/g) were identified as the predominant constituents [33]. However, the punicalagin content in the pericarp extracts from three Pakistani pomegranate samples obtained with 50% methanol was lower (98.7–118.6 mg/g) [34]. A study conducted on two Indian samples demonstrated that methanol was the most effective solvent for extracting ellagic and gallic acids, yielding up to 32.2 and 16.4 mg/g, respectively [35]. Hexane extraction resulted in the lowest recovery of these polyphenols, with maximum contents of 8.4 and 5.4 mg/g, respectively. Furthermore, the study reported significantly lower amounts of punicalagins, with methanol extracting up to 15.2 mg/g, whereas hexane was unable to recover these ellagitannins. Ethanolic pericarp extracts of the pomegranate samples collected in China [36] contained as much as 690 mg/g of punicalagins, along with 29.3 mg/g of ellagic acid and 25.3 mg/g of gallic acid. However, another study included 14 pomegranate samples collected in China revealed that pericarp extracts obtained ultrasonically using 40% ethanol contained between 39.8 and 121.5 mg/g of punicalagin isomers [25].

### 3.2. Antioxidant Activity

A comparison of the obtained values with those reported in the literature is challenging, as different methods and standard compounds have been used. Furthermore, the absence of standardization in the results frequently engenders contentious interpretations of antioxidant power. However, a substantial discrepancy in the activity of pomegranate pericarp extracts compared to juice has been documented in other studies [37]. In addition, research suggests that solvents with lower polarity tend to extract smaller quantities of phenolic compounds. Consequently, these extracts exhibit a reduced ability to scavenge free radicals [11,38].

Remarkable activity (IC_50_ = 18.63 µg/mL) was observed in the juice extract obtained from the Montenegrin cultivar sample using ethyl acetate with 1% isopropanol. In comparison to other juice extracts, which exhibited weak or moderate activity, this specimen can be regarded as an exception, likely attributable to its elevated gallic acid content, which is nearly 20 times higher than the lowest value observed in these extracts. The wild pomegranate extract obtained with ethyl acetate exhibited strong activity (IC_50_ = 25.66 µg/mL), which may be attributable to its high content of ellagic acid. In the absence of data regarding the type of extract obtained using the extraction technique employed in this study, a comparison of the results with those from the literature appears to be inconsistent. However, pomegranate juice itself is well-known for its strong antioxidant activity [39,40]. Some authors have highlighted the superiority of pomegranate juice over red wine and green tea, both of which are widely recognized as effective antioxidants. These authors have also demonstrated that industrially produced pomegranate juices exhibit superior antioxidant activity in comparison to laboratory-prepared juices. This observation underscores the importance of the juicing process and the pericarp-derived ellagitannins, which are considered to be one of the most responsible components for the antioxidant properties of pomegranate juice [26,41]. With respect to the IC_50_ assessed by DPPH, the literature contains a range of reported values. For instance, a value of 8.18 µg/mL was recorded for the juice of Tunisian origin [42], while an IC_50_ of 54.24 µg/mL was found for the juice derived from Romanian material [38]. The lyophilized juice, which is rich in punicalagins and ellagic acid, exhibited an IC_50_ value of 23 µg/mL [43]. Furthermore, a study conducted on the seed sarcotesta extracts reported DPPH IC_50_ values ranging from 10.12 to 112.32 µg/mL, indicating that the results were significantly influenced by extraction procedure and utilized solvent. The combination of ethanol, water, and diethyl ether yielded the most favorable yields. The most potent extracts contained significant amounts of ellagic acid (up to 34.5 mg/g) and gallic acid (up to 3.37 mg/g) [17]. With regard to the FRAP results, the values reported in the literature ranged from 17.65 to 47.1 mmol Fe^2+^/L [44]. As a reference, a recent study [45] involving wine extracts obtained using the same methodology as presented here demonstrated that diethyl ether extracts exhibited superior activity in comparison to ethyl acetate extracts. The DPPH IC_50_ and FRAP values for the red wine extracts obtained with diethyl ether ranged from 31.15 to 77.80 µg/mL and from 25.89 to 43.52 mmol Fe^2+^/g, respectively, demonstrating significantly better activity than the same extracts of pomegranate juice (153.45–230.41 µg/mL; 2.49–5.09 mmol Fe^2+^/g). In contrast, lower activity was reported for the red wine extracts obtained using ethyl acetate (75.49–105.1 µg/mL; 17.29–25.36 mmol Fe^2+^/g) compared to the same extracts in the present study (25.66–51.78 µg/mL; 8.52–19.41 mmol Fe^2+^/g) (Table 4).

A thorough examination of the results obtained from the pericarp extracts in this study reveals no significant differences between the extracts obtained through separate and successive Soxhlet extractions. However, it is evident that there is a modest decline in potency with successive extractions, likely due to the depletion of plant material resulting from repeated extraction processes. The ethyl acetate extracts obtained from the Italian cultivar sample demonstrated the lowest activity (DPPH IC_50_ 10.85 and 11.81 µg/mL; FRAP 26.16 and 26.23 mmol Fe^2+^/g). Conversely, the Montenegrin cultivar sample exhibited the highest activity (DPPH IC_50_ 3.34 and 5.17 µg/mL; FRAP 44.32 and 49.46 mmol Fe^2+^/g). The variation in IC_50_ values was particularly pronounced for PE19 and PE20, with a margin of more than threefold in some instances. A consideration of the phenolic compound content in these extracts and their related activity reveals the enhancing impact of gallic and ellagic acids. The extracts obtained from cultivar and wild Montenegrin samples were richer in their content than those from Italian samples. These differences were observed to be as high as twofold for ellagic acid (e.g., PE19 and PE21) and threefold for gallic acid (e.g., PE7 and PE8). Conversely, the punicalagins appear to have exhibited an antagonistic effect, as evidenced by the samples with elevated levels of these ellagic tannins, which demonstrated a modest decrease in activity when compared to PE20 and PE21 (compare with samples PE8 and PE9). With regard to the methanol extracts, the cultivated pomegranate from Italy exhibited the lowest activity, as indicated by its DPPH IC_50_ values of 4.12 and 4.73 µg/mL and its FRAP values of 155.83 and 143.51 mmol Fe^2+^/g. However, in this case, the wild pomegranate of Montenegrin origin exhibited the strongest antioxidant capacity, with a DPPH IC_50_ value almost three times lower (1.43 and 1.63 µg/mL) and more than a twofold difference in the FRAP values (372.17 and 315.86 mmol Fe^2+^/g). Given the content of phenolic compounds in methanol extracts, it is inevitable that punicalagin isomers had the most significant effect, thereby confirming earlier conclusions that they are the primary contributors to the antioxidant activity. The content of the former was found to be up to 40 times higher than that of the latter, which were obtained using ethyl acetate (PE22 and PE19). Methanol also demonstrated efficacy in extracting punicalin (up to 5.73 mg/g), given that other extracts generally contained negligible amounts of this ellagitannin. However, an exception was observed in the ethyl acetate extract PE20, which contained as much as 9.22 mg/g of punicalin. On the basis of the content and the activity of these extracts, it can be posited that they may exert positive or enhancing effects. Conversely, methanol exhibited reduced efficacy in extracting gallic and ellagic acids in comparison to ethyl acetate, resulting in a maximum decrease of up to fourfold in the content of these polyphenolic compounds (e.g., PE20 and PE23). Therefore, it appears to be an unreliable endeavor to definitively estimate their influence on the remarkable antioxidant activity reported for these extracts, particularly in the context of the predominance of punicalagin.

A comprehensive review of the available literature has yielded a substantial body of data concerning the antioxidant activity of pomegranate pericarp extracts. For instance, the DPPH IC_50_ value for the ethanol (70%) extract obtained from material originating from Bosnia and Herzegovina was determined to be 13.26 µg/mL [46]. The extracts derived from pomegranate samples from Portugal exhibited IC_50_ values ranging from 21 to 196 µg/mL, depending on the concentration (25%, 50%, or 75%) of ethanol used as the solvent [47]. However, lower values were recorded for the material originating from the USA (5.61–9.41 µg/mL) extracted with 50% ethanol [11]. The study also incorporated the methanol extract, which demonstrated an IC_50_ value of 5.03 µg/mL, aligning closely with the findings of the present study (1.43–4.73 µg/mL). Conversely, the methanol (50%) extract of the Iranian material demonstrated an activity with an IC_50_ value of 54 µg/mL [21]. Analogous outcomes were attained with the extracts of the Turkish-origin material (56–99 µg/mL, contingent on the methanol concentration employed as an extraction solvent) [37]. As reported by some authors, the IC_50_ values for the methanol and ethyl acetate extracts were 14.75 and 25.86 µg/mL, respectively [48]. Furthermore, other researchers found no detectable radical scavenging activity against DPPH for the extract obtained using ethyl acetate [49]. A review of the results of this study indicates that there is a significant increase in activity observed for the ethyl acetate extracts, with reported values ranging from 3.34 to 11.81 µg/mL.

A review of the FRAP literature reveals a value of 12.4 mmol Fe^2+^/g for the ethanol extract obtained from Palestinian material [50]. The aforementioned study on Bosnian material [46] reported a value of 7.137 mmol Fe^2+^/g, which was also observed for the ethanol extract. Another study involving the Jordan-origin material found values ranging from 0.86 to 1.11 mmol Fe^2+^/g, depending on the extraction solvent used, including methanol [49]. A thorough examination of the results from the present study reveals a conspicuously exceptional activity, particularly in the case of methanol extracts, where the FRAP value attained 372.17 mmol Fe^2+^/g, thereby signifying a noteworthy degree of potency.

### 3.3. Antimicrobial Activity

The analyzed extracts demonstrated a pattern of microbiological activity that was both pathogen- and composition-dependent. A consideration of the chemical composition of the extracts active against both CRAB and MSSA/MRSA (PE10, PE23, and PE24) reveals a high content of *α* and *β* punicalagin. This finding suggests that these compounds are likely responsible for the observed antimicrobial effects. This finding aligns with the established role of punicalagin, a naturally occurring compound found in pomegranate pericarp extracts, in exhibiting antimicrobial properties, particularly under its *α* and *β* anomeric forms [51]. In addition, punicalagin has been demonstrated to demonstrate efficacy against *S. aureus* by inducing structural alterations in the cell membrane [52]. Conversely, extracts demonstrating activity primarily against CRAB (JE2 and JE3) exhibited a predominance of gallic and ellagic acids. PE8, which demonstrated activity against MSSA and MRSA, contained a mixture dominated by ellagic acid, along with *α* + *β* punicalagin and gallic acid. A comparable, albeit less pronounced, composition was observed for JE6, which also demonstrated activity against MSSA and MRSA. The robust antibacterial activity of gallic acid has been exhaustively documented in the literature [53], thereby substantiating its potential contribution to the observed effects in the present study.

A comparison of the various types of extracts in terms of their solvent of choice reveals that only the methanolic (PE23 and PE24) and ethyl acetate (PE8 and PE21) extracts of pomegranate pericarp demonstrated significant activity. The low yields of hexane- and dichloromethane-derived extracts constrained the number that could be evaluated; nevertheless, no antimicrobial activity was detected for these extracts (PE14 and PE17). For juice extracts, the most active sample was identified as JE6, which was prepared using ethyl acetate. The present findings are consistent with the earlier discussion on the influence of extraction solvents on the recovery of bioactive compounds.

Although the antibacterial activity of pomegranate against various microorganisms has been previously reported, to the best of the authors’ knowledge, no studies have specifically addressed its activity against multidrug-resistant Gram-negative pathogens, such as carbapenem-resistant bacteria. In this context, high activity was observed for certain extracts against CRAB, a well-recognized nosocomial pathogen noted for its biofilm formation on medical devices and prolonged survival in hospital environments [54]. The observed high sensitivity of *S. aureus* strains and marked resistance of *E. coli* are consistent with some previous reports [55,56]. The methanolic extract from the pericarp of the Palestinian pomegranate demonstrated bactericidal properties against *S. aureus* and *K. pneumoniae*, with minimum bactericidal concentrations (MBCs) of 1.25% and 2.5%, respectively, while exhibiting no activity against *E. coli* [57]. Conversely, the aforementioned study documented robust fungicidal activity against CA (MBC = 0.078% *p*/*v*), a finding that stands in contrast to the outcomes observed in the present study. It has been documented in other studies that the minimum bactericidal concentration (MBC) values for pericarp extracts range from 2.5 to 5% *p*/*v*, with the MBC values demonstrating effectiveness against Gram-negative bacteria, such as *Escherichia coli*, as well as Gram-positive bacteria, including *Staphylococcus aureus* and *Klebsiella pneumoniae* [58]. Due to the absence of directly comparable data for pomegranate juice extracts in the literature, a meaningful comparison with the results presented in this study could not be performed. Nonetheless, the literature pertaining to the activity of pomegranate juice (or its preparations) suggests the presence of moderate to strong antimicrobial effects [59], albeit with the caveat that the current corpus of research is still limited. For instance, lyophilized juice has been reported to exhibit antibacterial activity against various *K. pneumoniae* strains, with an MBC of 0.1024% *p*/*v* [60].

## 4. Materials and Methods

### 4.1. Plant Material

Three different pomegranate fruit samples were selected for this study: two cultivars originating from Montenegro and Italy, purchased from local markets, and a wild sample collected in Stijena Piperska (42.511800° N, 19.257398° E) in Montenegro. Initially, whole fruits were subjected to mechanical compression using a press, thereby yielding fresh juice samples. These samples were then promptly utilized for the extraction process. The remaining pericarps (peels/rinds) were expeditiously separated and subjected to air-drying at ambient temperature for a period of 25 days. Prior to extraction, the samples of the dried pericarp were ground in a blender.

### 4.2. Liquid–Liquid Extraction

For the liquid–liquid extraction process, the Kutscher-Steudel extractor was employed. The apparatus was designed for the extraction of aqueous solutions using solvents lighter than water, originally diethyl ether, though other solvents may be used. A precise volume of the sample is meticulously transferred into the extraction flask, while the solvent, contained within a separate flask, is gradually heated to a temperature that exceeds its boiling point. The resulting vapor is then directed towards a reflux condenser, where it undergoes condensation. Thereafter, the vapor is channeled through a funnel, ultimately reaching the base of the extraction flask. At this juncture, the vapor comes into contact with the sample. The solvent diffuses through the sample due to its disparate densities, thereby dissolving its bioactive compounds. The enriched solvent then returns to the boiling flask, where the cycle is repeated, ensuring continuous extraction [45,61,62].

Here, 70 mL of juice were transferred into a 100 mL extraction flask. The juice sample was subsequently covered with a sufficient amount of the selected solvent until it reached the outlet of the flask. A quantity of 150 mL of the solvent was transferred into a 250 mL receiving flask and subsequently heated to its boiling point. Over the course of a 24 h period, the solvent was found to become enriched with juice components. The solvents employed in this study included diethyl ether, ethyl acetate, and 1% (*v*/*v*) isopropanol in ethyl acetate. The extracts were subsequently obtained by subjecting the solution to a process of solvent removal, employing a rotary evaporator to facilitate the evaporation of the solvents. A total of nine final extracts were obtained (see Table 6). These extracts were stored at 4 °C in airtight containers until further use.

### 4.3. Solid–Liquid Extraction

Soxhlet extraction is a continuous solid–liquid extraction that combines elements of both multiple percolation and maceration. The process is carried out using a Soxhlet apparatus, which operates on the principles of solvent reflux and siphoning. These methods are employed to efficiently extract bioactive compounds with a pure solvent [63]. The technique has been recognized as the gold standard for the extraction of compounds, particularly phenols, from solid matrices. It serves as a reference for the evaluation of other extraction methods. The sample is ground prior to extraction in order to increase the area of solid–liquid contact. Subsequently, the sample is placed in a filter paper holder within the extractor. The lowermost extremity of the extractor is affixed to a round bottom flask containing a solvent, and it is linked to a reflux condenser. The solvent contained within the flask is subjected to an increase in temperature until it reaches its boiling point. Concurrently, vapor begins to rise through the branch pipe of the extractor. These vapors subsequently condense and drip into the extractor, where they come into contact with the sample, thereby facilitating extraction. Once the solvent level exceeds the highest point of the siphon, the solvent (which is now enriched with the extracted compounds) is siphoned back into the flask. The process is characterized by its continuous nature, ensuring that fresh solvent repeatedly interacts with the sample while the extracted compounds accumulate in the flask [64]. The recurrent phenomenon of siphoning is designated as a “cycle.” The duration of the complete extraction process is variable, and its measurement is typically expressed in hours or cycles. Consequently, the method is frequently adapted or modified.

In this study, extractions were performed using 20 g of plant material (dried pericarp) from each pomegranate sample. The extraction process involved the use of 250 mL of hexane, dichloromethane, ethyl acetate, and methanol as the solvent, with the order of extraction being as follows: hexane, dichloromethane, ethyl acetate, and methanol. Each extraction was conducted for a duration of 24 h, yielding a total of 12 extracts (see Table 6 for details). However, the majority of the extracts obtained with hexane and dichloromethane were deemed inadequate and were consequently excluded from further analysis.

In addition to the aforementioned individual extractions, continuous Soxhlet extractions were performed, employing 70 g of plant material and adding solvents successively in the previously specified order. Each extraction was conducted for a duration of 24 h. This modification enabled the sequential extraction of the same plant material with different solvents, one after the other. Consequently, 12 extracts were obtained (see Table 6). It should be noted that the first two solvents (hexane and dichloromethane) yielded insufficient extracts for further analyses.

### 4.4. Determination of Individual Phenolic Compounds

The identification of compounds present in pomegranate samples was conducted using a 1200 HPLC system (Agilent Technologies, Santa Clara, CA, USA) equipped with a Synergy TM Hydro-RP 80 Å (4 µm, 150 × 4.6 mm) column. The analysis utilized two mobile phases: phase A, a 0.1 M solution of phosphoric acid, and phase B, pure acetonitrile. The flow rate was measured to be 0.800 mL/min, with photodiode-array (PDA) detection (Agilent Technologies, Santa Clara, CA, USA) (UV at 260 nm) consistently maintained within a 45 min time frame. The optimal peak separation was attained through the implementation of the following combination: 2% B (0 min); 2–10% B (0–5 min); 10% B (5–15 min); 10–15% B (15–20 min); and 15–60% (20–40 min). Prior to injection, the extracts under investigation (all samples were in a concentration range of 10 to 20 mg per mL of methanol) were filtered through a polyethylene terephthalate (PET) membrane filter. The injection volume of standard solutions and the tested extract was 5 µL. The identification process was conducted through the utilization of overlay curves and retention times. Following the successful implementation of the spectra matching process, the outcomes were substantiated through the utilization of spiking with respective standards. This approach was employed to attain comprehensive identification, which was subsequently evaluated through the implementation of the so-called peak purity test. The peaks that did not satisfy these criteria were not quantified. The quantification process was executed by implementing external calibration using a standard. Triplicate measurements were taken, and the resulting data were presented as the mean ± standard deviation (SD). The utilized concentrations of the reference substances were 0.051, 0.198, 0.114, 0.126, and 0.043 mg/mL for gallic acid, *p*-hydroxycinnamic acid, punicalin, punicalagin, and ellagic acid, respectively.

The “fingerprinting” of the investigated anthocyanin was achieved by means of an Agilent Technologies 1200 HPLC system equipped with a Lichrospher 100RP 18e column. This system was configured to apply gradient elutions of two mobile phases, designated “A/B” (0.2 M phosphoric acid solution “A” and pure acetonitrile “B”). The flow rate was set at 1 mL/min, and the photodiode array (PDA) detection was performed using UV at 260 and 325 nm. Each analysis was completed within a duration of 70 min. The optimal combinations were identified as 89–75% A (0–35 min); 75–60% A (35–55 min); 60–35% A (55–60 min); and 35–0% A (60–70 min). Prior to injection, the samples were filtered through a PTFE membrane filter (Agilent Technologies, Santa Clara, CA, USA). The identification process was conducted through the utilization of overlay curves and retention times. Following the successful implementation of the spectra matching process, the outcomes were validated through the utilization of spiking with corresponding standards, thereby facilitating comprehensive identification via the designated peak purity test. The peaks that did not satisfy these criteria were not quantified. Quantification was performed by employing external calibration with standards. Triplicate measurements were taken, and the resulting data were presented as the mean ± standard deviation (SD). The concentrations of the utilized standards were determined to be 0.416 mg/mL for cyanidin chloride and 0.356 mg/mL for malvidin-3-*O*-glucoside, respectively.

### 4.5. Determination of Antioxidant Activity

To evaluate the antioxidant activity, DPPH and FRAP assays were carried out on the obtained pomegranate fruit extracts. Prior to analysis, stock solutions (1 mg/mL) were prepared by thoroughly dissolving the extracts in methanol.

#### 4.5.1. DPPH Assay

The free radical scavenging activity of the extracts was measured by the DPPH method [65]. In summary, a solution of DPPH with a concentration of 0.1 mM was prepared in methanol. A volume of 3 mL of the extract of the appropriate dilution and 1 mL of the DPPH solution were mixed in a test tube. The mixture was vigorously agitated and subsequently left undisturbed in a dark environment at room temperature for a period of 30 min. Subsequently, the absorbances were measured at a wavelength of 517 nanometers (nm), corresponding to the maximum absorption peak of the sample. Ascorbic acid was utilized as a reference standard. The experiment was performed in triplicate. The percent DPPH scavenging effect (RSA) or inhibition percentage of antioxidant activity (%AA) was calculated by the following equation:

$$\%AA=\frac{{(A}_{0}-A_{1})}{A_{0}}\times 100$$

In the experimental design, A_0_ represented the control’s measured absorption, while A_1_ corresponded to the absorption of the extract or the standard solution. The IC_50_ (the concentration required to inhibit 50% of the DPPH free radical; µg/mL) value of the extracts or ascorbic acid was calculated using log-dose inhibition curve; a lower IC_50_ value indicates a higher antioxidant activity. In order to perform an adequate assessment of antioxidant strength, the antioxidant activity index (AAI) was calculated according to the following formula:

$$AAI=\;\frac{DPPH\;final\;concentration\;mg/mL}{{IC}_{50}\;mg/mL}$$

Consequently, the antioxidant strength is regarded as weak when the AAI is less than 0.5, moderate when it is between 0.5 and 1, high when it ranges from 1 to 2, and very strong when the AAI is more than 2 [66,67].

#### 4.5.2. FRAP Assay

The FRAP test was used to determine the reducing capacity of the extracts [68]. First, the FRAP reagent was prepared by mixing 300 mM sodium acetate buffer solution at pH 3.6, 10 mM 2,4,6-tripyridyl-s-triazine (TPTZ) diluted in 40 mM hydrochloride acid and 20 mM ferric chloride in a ratio of 10:1:1, respectively. Subsequently, the reaction mixture was prepared by adding 3 mL of ferric-reducing antioxidant power (FRAP) reagent, 1 mL of distilled water, and 100 microliters of each extract stock solution. The tubes were then subjected to an incubation period of 15 min at a temperature of 37 °C. Subsequently, the tubes were permitted to stand at room temperature for an additional five minutes, after which the absorbances were measured at a wavelength of 595 nanometers (nm). From the calibration curve of ferrous sulfate heptahydrate, utilized as a standard (0–1000 µmol/L), antioxidant capacity values were determined and expressed as mmol Fe^2+^ equivalents per g of extract dry weight. It has been demonstrated that an increase in the Fe^2+^ concentration is concomitant with an increase in the ferric-reducing antioxidant power.

### 4.6. Determination of Antimicrobial Activity

The antimicrobial activity of pomegranate extracts was determined using the following microorganisms: methicillin-susceptible *S. aureus* [MSSA (ATCC 29213)], *E. coli* (ATCC 25922), *C. albicans* (ATCC 14053), methicillin-resistant *S. aureus* [MRSA (isolated clinical strain)], carbapenem-susceptible *K. pneumoniae* (isolated clinical strain), carbapenem-resistant *K. pneumoniae* (isolated clinical strain), and carbapenem-resistant *A. baumannii* (clinical strain). The clinical isolates used were identified and characterized by routine standard clinical microbiology methods.

Until further analyses could be conducted, the microorganisms were stored on a cryovial bead preservation system (Microbank; Pro-Lab Diagnostics, Richmond Hill, ON, Canada) at −80 °C. The inoculum was prepared by spreading one cryovial bead on a blood agar plate and subsequently incubating it overnight at a temperature of 37 °C. One colony was re-suspended in 5 mL of tryptic soy broth (TSB) and subsequently incubated at 37 °C without shaking. Subsequently, overnight cultures were adjusted to a turbidity of 0.5 McFarland, corresponding to approximately 1 × 10^8^ colony-forming units (CFU) per mL.

The extracts were dissolved in distilled water. The determination of minimal bactericidal concentrations (MBCs) was only possible due to the opalescent nature of the initial solution. Cation-adjusted Mueller Hinton (CAMHB) and Sabouraud broth were utilized for the isolation of bacteria and fungi, respectively. In summary, two-fold serial dilutions of extracts were prepared in 2 mL Mueller Hinton broth (MHB) in borosilicate glass tubes and incubated for 18 h at 37 °C. Bactericidal activity was defined as a minimum of 99.9% (i.e., a minimum of 3-log10 CFU/mL) reduction in the initial bacterial count at each designated time point. The initial bacterial inoculum was estimated to be approximately 5 × 10^5^ colony-forming units (CFU) per mL. The MBC value was expressed as a percentage of the weight over the volume (% *p*/*v*).

## 5. Conclusions

This study demonstrates the high functional potential of pomegranate juice and pericarp extracts. It also highlights the influence of solvent polarity on the recovery of bioactive phenolic compounds. Pericarp extracts, which are abundant in ellagitannin punicalagin isomers, demonstrated remarkably robust antioxidant and substantial antimicrobial properties. These findings indicate their potential as natural agents against antibiotic-resistant bacterial strains. Furthermore, juice extracts, obtained via liquid–liquid extraction, exhibited significant bioactivities, suggesting their potential as functional ingredients. The results obtained lend support to the valorization of both juice and pericarp for applications in pharmacy and cosmeceuticals. Furthermore, they provide a foundation for further research on optimizing extraction methods and understanding the specific contributions of individual phenolic compounds to bioactivity.

## Figures and Tables

**Table 1:** Pomegranate juice extracts yields.

Extract ^1^	Solvent	Origin ^2^	Yield (g/100 mL) ^3^
JE1	diethyl ether	Italy	0.64
JE2		Montenegro	1.26
JE3		Montenegro	2.58
JE4	ethyl acetate	Italy	3.56
JE5		Montenegro	2.64
JE6		Montenegro	3.61
JE7	1% (*v*/*v*) isopropanol in ethyl acetate	Italy	3.95
JE8		Montenegro	3.21
JE9		Montenegro	6.26

^1^ Extract names (JE1–JE9) correspond to the extracts described in Section 4; ^2^ Both cultivated and wild fruits were used, as detailed in Section 4; ^3^ Values are expressed as grams of extract per 100 mL of juice sample; ash content was not determined for these samples.

**Table 2:** Pomegranate pericarp extracts yields.

Extract ^1^	Solvent ^2^	Origin ^3^	Yield (g/100 mL) ^4^
PE1	hexane	Italy	0.21
PE2		Montenegro	0.21
PE3		Montenegro	0.19
PE4	dichloromethane	Italy	0.46
PE5		Montenegro	0.35
PE6		Montenegro	0.38
PE7	ethyl acetate	Italy	6.12
PE8		Montenegro	8.39
PE9		Montenegro	6.26
PE10	methanol	Italy	49.55
PE11		Montenegro	63.51
PE12		Montenegro	25.21
PE13	hexane	Italy	0.16
PE14		Montenegro	0.32
PE15		Montenegro	0.19
PE16	dichloromethane	Italy	0.26
PE17		Montenegro	0.21
PE18		Montenegro	0.20
PE19	ethyl acetate	Italy	3.70
PE20		Montenegro	5.17
PE21		Montenegro	5.97
PE22	methanol	Italy	29.68
PE23		Montenegro	44.36
PE24		Montenegro	37.12

^1^ Extract names (PE1–PE24) correspond to the extracts described in Section 4; ^2^ Both separate and successive Soxhlet extractions were used, as detailed in Section 4; ^3^ Both cultivated and wild fruits were used, as detailed in Section 4; ^4^ Values are expressed as % (*w*/*w*) of dried pericarp sample; ash content was not determined for these samples.

**Table 3:** Polyphenolic compounds content of pomegranate fruit extracts analyzed.

Extract Name ^1^	Polypenolic Compound ^2^						
	Gallic Acid	*p*-Hydroxycinnamic Acid	Punicalin	*α* + *β* Punicalagin	Ellagic Acid	Cyanidin Chloride	Malvidin-3-*O*-glucoside
JE1	0.29	0.04	-	1.11	0.56	-	-
JE2	0.50	-	-	0.42	0.81	-	0.06
JE3	0.62	-	-	0.31	0.82	0.04	-
JE4	0.21	1.2	0.35	10.33	1.92	-	-
JE5	0.78	0.93	-	2.47	2.31	-	-
JE6	0.96	-	0.29	3.34	4.07	0.23	-
JE7	0.20	1.4	0.45	11.71	1.70	-	-
JE8	3.81	-	-	0.23	1.89	-	-
JE9	0.84	0.97	-	1.13	2.55	-	-
PE2	0.13	-	0.26	2.26	0.46	-	-
PE3	-	2.23	-	0.14	0.32	-	-
PE5	0.12	-	0.27	4.92	1.25	-	-
PE6	0.09	-	0.21	7.10	1.13	-	-
PE7	2.49	1.26	-	9.44	15.34	-	-
PE8	7.68	-	0.79	18.81	27.97	-	-
PE9	3.42	-	0.62	21.81	20.83	-	-
PE10	1.25	-	4.69	217.88	14.65	-	-
PE11	2.96	-	5.62	207.58	8.03	-	-
PE12	2.68	-	5.72	254.75	13.56	-	-
PE13	2.68	0.23	0.54	-	0.14	-	-
PE15	0.21	4.47	-	0.89	1.13	-	-
PE16	0.11	9.93	2.87	0.5	0.18	-	-
PE18	-	1.03	1.52	3.58	0.47	-	-
PE19	2.96	1.15	-	5.90	15.78	-	-
PE20	9.21	-	9.22	6.86	21.51	-	-
PE21	6.43	-	0.28	8.22	30.03	-	-
PE22	1.40	1.44	1.96	248.07	7.03	-	-
PE23	2.05	-	4.52	228.34	6.38	-	-
PE24	2.59	-	4.99	214.92	12.09	-	-

^1^ JE1–JE9: juice extracts; PE2–PE24: pericarp extracts (see Section 4 for details); ^2^ Values are expressed as mg per g of dry weight of extract (mg/g); “-” indicates that the compound was not detected under the applied analytical conditions; ash content was not determined for these samples.

**Table 4:** Antioxidant capacities of the pomegranate extracts examined.

Extract Name	DPPH IC_50_ (µg/mL)	AAI	FRAP (mmol Fe^2+^/g)
JE1	230.41 ± 1.12	0.171	2.49 ± 0.07
JE2	170.41 ± 1.22	0.231	4.47 ± 0.08
JE3	153.45 ± 0.59	0.257	5.09 ± 0.84
JE4	51.78 ± 1.01	0.761	8.52 ± 0.63
JE5	48.52 ± 0.74	0.812	17.38 ± 0.85
JE6	25.66 ± 0.65	1.54	19.41 ± 0.69
JE7	43.03 ± 1.04	0.92	12.04 ± 0.92
JE8	18.63 ± 0.64	2.12	18.69 ± 1.03
JE9	43.38 ± 2.78	0.91	15.55 ± 0.86
PE1	218.73 ± 2.89	0.18	3.17 ± 0.83
PE5	199.81 ± 0.95	0.19	3.45 ± 0.45
PE7	10.85 ± 0.78	3.63	26.16 ± 1.11
PE8	5.17 ± 0.91	7.63	49.46 ± 1.32
PE9	7.54 ± 0.51	5.23	37.46 ± 0.66
PE10	4.12 ± 0.13	9.56	155.83 ± 1.95
PE11	2.86 ± 0.45	13.78	162.56 ± 0.98
PE12	1.43 ± 0.59	27.57	372.17 ± 0.83
PE18	210.71 ± 0.84	0.18	2.99 ± 0.58
PE19	11.81 ± 0.75	3.14	26.23 ± 1.02
PE20	3.34 ± 0.55	10.14	44.32 ± 2.11
PE21	7.19 ± 0.75	4.96	47.26 ± 1.51
PE22	4.73 ± 0.65	7.33	143.51 ± 1.97
PE23	2.93 ± 0.15	12.79	152.06 ± 2.01
PE24	1.63 ± 0.51	18.42	315.86 ± 1.91
ascorbic acid	5.45 ± 0.78	7.22	n/a

Values represent mean ± SD of three independent measurements (*n* = 3); statistical comparisons among extracts were not performed, and the data are presented for comparative and descriptive purposes. JE = juice extract, PE = pericarp extract (see Section 4); IC_50_ = half maximal inhibitory concentration; AAI = antioxidant activity index; FRAP = ferric reducing antioxidant power; values are expressed on a dry weight (DW) basis, and ash content was not determined. “n/a” indicates not applicable.

**Table 5:** Antimicrobial activities of the pomegranate extracts examined; MBC values are expressed as % *p*/*v*.

Extract Name	Microorganism Tested						
	MSSA	MRSA	*E. coli*	Carbapenem Susceptible KPC	Carbapenem Resistant KPC	CRAB	CA
JE2	1.126	1.126	0.563	>1.126	1.126	0.308	>1.126
JE3	1.011	1.011	1.011	>1.011	>1.011	0.252	>1.011
JE5	1.604	0.716	1.432	1.604	1.604	1.604	1.604
JE6	0.159	0.159	0.638	>0.638	>0.638	0.638	>0.638
JE8	1.611	1.611	0.805	1.611	1.611	0.805	1.611
PE8	1.248	1.248	>2.496	>2.496	>2.496	1.248	>2.496
PE10	1.271	1.271	2.543	>2.543	>2.543	0.635	>2.543
PE14	>0.408	>0.408	>0.408	>0.408	>0.408	>0.408	>0.408
PE17	>0.272	>0.272	>0.272	>0.272	>0.272	>0.272	>0.272
PE20	3.640	3.640	3.640	3.640	3.640	3.640	>3.640
PE21	0.952	>1.904	1.904	>1.904	>1.904	1.904	>1.904
PE23	0.337	0.168	1.262	>2.698	0.674	0.674	>2.698
PE24	0.109	0.109	0.877	>0.877	>0.877	0.109	>0.877

MBC = minimum bactericidal concentration; JE = juice extract, PE = pericarp extract (see Section 4); values are expressed on a dry weight (DW) basis, and ash content was not determined. MSSA = methicillin-susceptible *Staphylococcus aureus*; MRSA = methicillin-resistant *S. aureus*; KPC = *Klebsiella pneumoniae* clinical strain; CRAB = carbapenem-resistant *Acinetobacter baumannii*; CA = *Candida albicans*. Statistical comparisons among extracts were not performed; the data are presented for comparative and descriptive purposes. “>” indicates no bactericidal activity detected at the tested concentration.

**Table 6:** A total of 33 pomegranate fruit extracts obtained with liquid–liquid and solid–liquid extraction processes; the solvent for extraction, fruit part, sample, origin and extract name are listed.

Solvent	Fruit Part	Type of Extraction		Cultuvar ^1^ Italy	Cultivar Montenegro	Wild Montenegro
diethyl ether	juice	liquid–liquid (Kutscher-Steudel)		JE1 ^2^	JE2	JE3
ethyl acetate				JE4	JE5	JE6
1% (*v*/*v*) isopropanol in ethyl acetate				JE7	JE8	JE9
hexane	pericarp	solid–liquid (Soxhlet)	separate	PE1 ^3^	PE2	PE3
dichloromethane				PE4	PE5	PE6
ethyl acetate				PE7	PE8	PE9
methanol				PE10	PE11	PE12
hexane			successive	PE13	PE14	PE15
dichloromethane				PE16	PE17	PE18
ethyl acetate				PE19	PE20	PE21
methanol				PE22	PE23	PE24

^1^ Three pomegranate fruit samples were used as it is reported in Section 4.1; ^2^ JE—juice extract; ^3^ PE—pericarp extract.

## Data Availability

All data generated or analyzed during this study are included in this published article.
